# Interactive effects of incentive value and valence on the performance of discrete action sequences

**DOI:** 10.1038/s41598-021-88286-5

**Published:** 2021-04-29

**Authors:** Tyler J. Adkins, Bradley S. Gary, Taraz G. Lee

**Affiliations:** Department of Psychology, University of Michigan, Ann Arbor, MI USA

**Keywords:** Consolidation, Reward, Human behaviour, Cognitive control, Motivation, Long-term memory, Motor cortex, Premotor cortex

## Abstract

Incentives can be used to increase motivation, leading to better learning and performance on skilled motor tasks. Prior work has shown that monetary punishments enhance on-line performance while equivalent monetary rewards enhance off-line skill retention. However, a large body of literature on loss aversion has shown that losses are treated as larger than equivalent gains. The divergence between the effects of punishments and reward on motor learning could be due to perceived differences in incentive value rather than valence per se. We test this hypothesis by manipulating incentive value and valence while participants trained to perform motor sequences. Consistent with our hypothesis, we found that large reward enhanced on-line performance but impaired the ability to retain the level of performance achieved during training. However, we also found that on-line performance was better with reward than punishment and that the effect of increasing incentive value was more linear with reward (small, medium, large) while the effect of value was more binary with punishment (large vs not large). These results suggest that there are differential effects of punishment and reward on motor learning and that these effects of valence are unlikely to be driven by differences in the subjective magnitude of gains and losses.

When people are highly motivated, they perform with greater speed and accuracy. Motivation often arises from cues in the environment that signal the prospect of a performance-contingent reward or punishment. Accordingly, the scientific study of motivation in humans often examines the effects of performance-contingent monetary gains and losses on behavior and brain activity^1^. Motivation induced by monetary incentives influences the learning^2^, performance^3^, and long-term retention^4^ of skilled actions in humans.

The effects of motivation on skilled action depend on incentive size^5,6^ and incentive valence^7^. Compared to training with reward, some studies have found that training in a serial response time task with punishing feedback enhances performance *during* training but impairs later skill retention^2,4,8^. One possibility is that these observed differences between punishment and reward are driven by differences in the subjective magnitude of gains and losses. People often treat gains as having greater (dis)utility than equivalent losses in economic choice tasks, a phenomenon known as loss aversion^9^. If prior valence effects are driven by loss aversion, then the effects of valence (punishment – reward) should be in the same direction as effects of increasing value (e.g., $30—$5). In addition, the effects of increasing incentive value should be greater for punishment than reward. No study to our knowledge has examined interacting effects of the valence *and* value of incentives in the context of training skilled actions. So, it remains a possibility that valence effects are really just value effects.

To test this hypothesis, we conduct a study in which we manipulate both incentive value and incentive valence while participants learned motor sequences. In total, 93 human participants trained to perform motor sequences for rewards of varying size (Reward group), punishments of varying size (Punishment group), or no incentives (Control group). Consistent with our hypothesis, we found that large incentives enhanced on-line performance but impaired the ability to retain the level of performance achieved during training. However, we also found that on-line performance was better with reward than punishment and that the effect of increasing incentive value was more linear with reward (small, medium, large) while the effect of value was more binary with punishment (large vs not large). These results suggest that differential effects of punishment and reward on motor learning are not driven by mere differences in the subjective magnitude of gains and losses.

## Results

### Value-driven enhancements to movement speed were more linear for rewards

**Figure 1:**
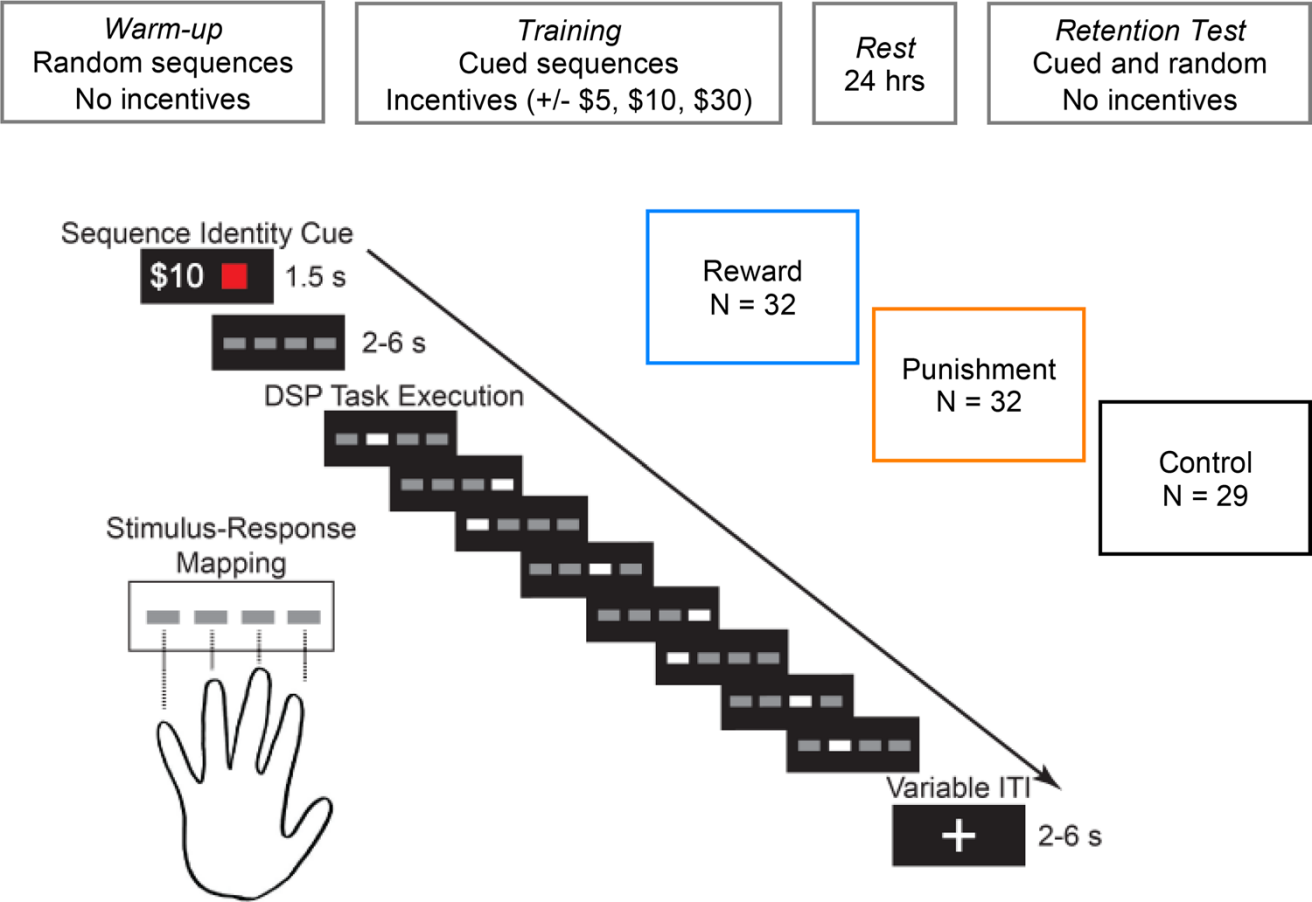
Experimental Design. Participants performed a discrete sequence production task with opportunities for monetary gains (reward) or losses (punishment). Each participant learned three unique 8-item sequences and each sequence was paired with an incentive of $5, $10, or $30. The control group performed the same task but without incentives. Each trial began with a cue signaling which sequence to perform (for all groups) and the size of the associated incentive (for incentive groups). Participants were instructed to perform the sequences as quickly and accurately as they could. Participants returned to the lab a day after training to test how well they retained their level of performance when incentives were no longer present.

**Figure 2:**
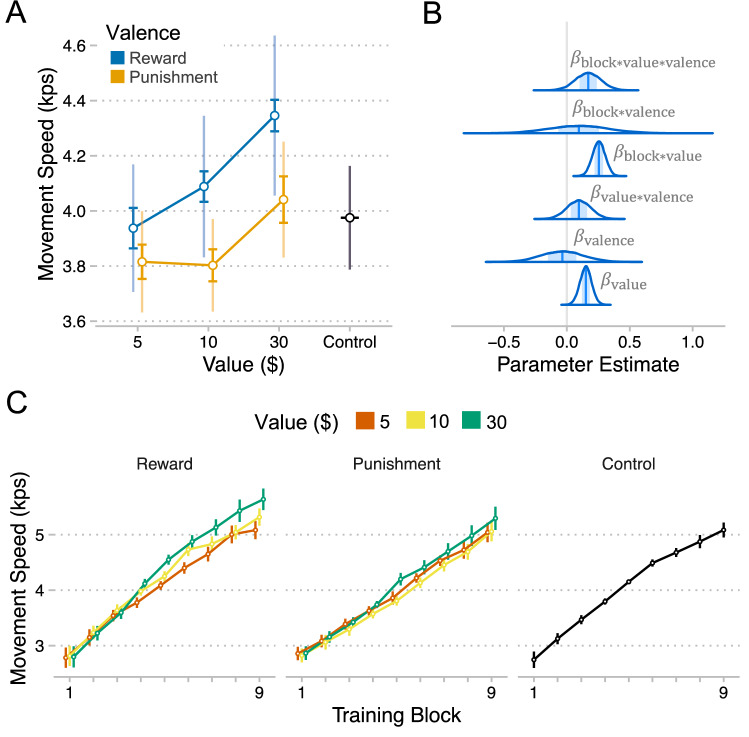
Effects of incentives on the speed of movement execution. (**A**) Speed (mean + /- se) for each level of value by valence. The bold error bars represent within-subject standard error (for value effect) and the non-bold error bars are between-subject standard error (for valence effect). (**B**) Densities of MCMC samples used to approximate posterior probability distributions over key regression coefficients. C. Speed for each level of block by value (sequence) by valence (group). Plots were created using ggplot2^46^.

Do incentives enhance the speed with which people execute movement sequences? We address this question by fitting a gaussian regression model to participants’ movement speeds (speed) at each level of block by value and by valence (Figs. 1, 2B; See Methods for modeling details). Our participants performed sequences more quickly over the course of training, as evidenced by a positive linear effect of block on speed ($\beta = 1.44, CI = \left[ {1.20,1.68} \right], P_{d} < .001$) (Fig. 2C). There was also a negative *quadratic* effect of block ($\beta = - 0.08, CI = \left[ { - 0.18, 0.01} \right], P_{d} = .05$), suggesting that performance improvements were more pronounced at the start of training. Pairing sequences with higher value incentives enhanced movement speed and the effect of training on movement speed, as shown by a positive effect of incentive value on speed ($\beta = 0.15, CI = \left[ {0.06, 0.24} \right], P_{d} = .001$) and a positive interaction between incentive value and block ($\beta = 0.26, CI = \left[ {0.16, 0.35} \right], P_{d} < .001$), respectively.

We found no evidence for a main effect of valence ($$\beta = - 0.03, CI = \left[ { - 0.37, 0.29} \right], P_{d} = .43$$), however there was evidence that the value effect and the block by value interaction were greater for the Reward compared to Punishment (value*valence: $$\beta = 0.10, CI = \left[ { - 0.09, 0.28} \right], P_{d} = .15$$; block*value*valence: $$\beta = 0.17, CI = \left[ { - 0.03, 0.37} \right], P_{d} = .05$$). This is confirmed by fitting the separate models to each group, which showed that value-driven enhancement was more pronounced for Reward (value: $$\beta = 0.19, CI = \left[ {0.07, 0.33} \right], P_{d} < .001$$; value*block: $$\beta = 0.34, CI = \left[ {0.19, 0.48} \right], P_{d} < .001$$) compared to Punishment (value: $$\beta = 0.10, CI = \left[ { - 0.06, 0.24} \right], P_{d} = .09$$; value*block: $$\beta = 0.17, CI = \left[ {0.04, 0.30} \right], P_{d} = .01$$). We also found that movement speed in training was faster with large reward compared to control ($$\beta = 0.21, CI = \left[ { 0.04, 0.36} \right], P_{d} = .01$$), but not so with punishment or small reward ($30 Punishment: $$\beta = 0.03, CI = \left[ { - 0.11, 0.16} \right], P_{d} = .36;$$$5 Punishment: $$\beta = - 0.08, CI = \left[ { - 0.21, 0.05} \right], P_{d} = .12$$; $5 Reward: $$\beta = 0.00, CI = \left[ { - 0.14, 0.15} \right], P_{d} = .48$$). These results suggest that performance improvements during the training period were greatest for sequences paired with large rewards.

We next tested whether increasing incentive values had qualitatively distinct effects depending on whether they were framed as rewards or punishments. A model-comparison analysis using Punishment data demonstrated that a model with binary value coding predicted held-out data better than a model with linear value coding (binary – linear: $$M{ } = - 41.6, SE = 15.7$$) (Fig. 2A). However, for Reward, a linear value function led to better predictive accuracy (linear – binary: $$M{ } = - 34.0, SE = 15.7$$). It appears that participants in the Punishment group treated the high value ($30) as categorically distinct from other values, while participants in the Reward group did not and instead showed more linear improvements with increasing value.

### Value-driven enhancements to movement accuracy were more linear for rewards

**Figure 3:**
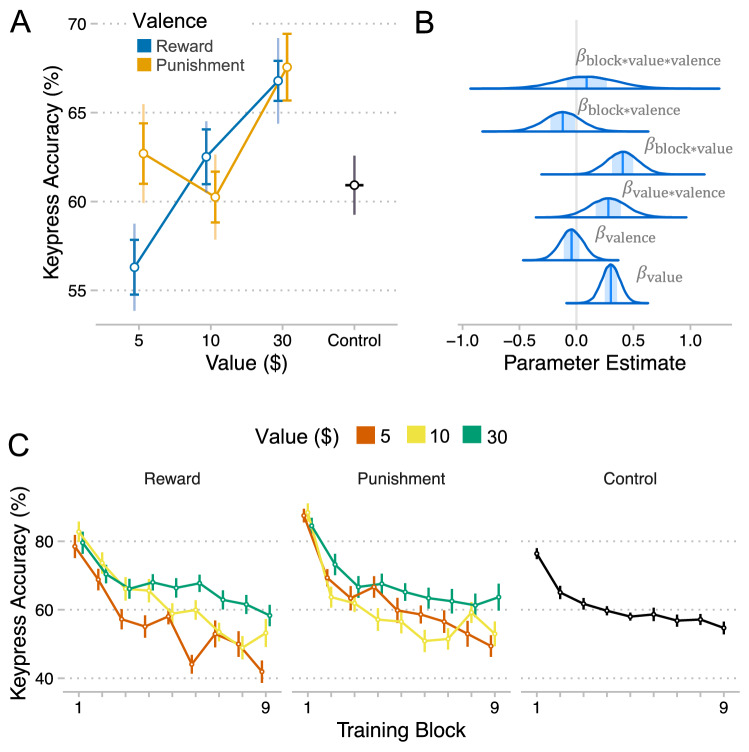
Effects of incentives on the accuracy of movement execution. (**A**) Accuracy (mean + /- se) for each level of value by valence. The bold error bars represent within-subject standard error (for value effect) and the non-bold error bars are between-subject standard error (for valence effect). (**B**) Densities of MCMC samples used to approximate posterior probability distributions over key regression coefficients. (**C**) Accuracy for each level of block by value (sequence) by valence (group). Plots were created using ggplot2^46^.

Increases in speed often beget decreases in accuracy, but does this tradeoff apply to incentive-motivated improvements in movement speed? We address this question by fitting a beta regression model to success rates (accuracy) at each level of block by value by valence (Fig. 3B). We measure accuracy according to whether the target sequence was completed without keypress errors under the specified time limit (See Methods for modeling details).The trajectory of accuracy through training mirrored the trajectory of movement speed, with a negative linear effect of block ($\beta = - 0.92, CI = \left[ { - 1.09, - 0.76} \right], P_{d} < .001$) and a positive quadratic effect of block ($\beta = 0.30, CI = \left[ {0.19, 0.42} \right], P_{d} < .001$) (Fig. 3C). In this case though, the decrease in accuracy over time is likely because our time limits created an imperative to strive for increased speed throughout training. However, the negative effect of block was less pronounced for higher value sequences, suggesting participants could maintain higher levels of accuracy on these sequences (block*value: $\beta = 0.41, CI = \left[ {0.14, 0.66} \right], P_{d} = .001$). Critically, we found a positive main effect of value on accuracy ($\beta = 0.30, CI = \left[ {0.14, 0.46} \right], P_{d} < .001$), demonstrating that accuracy improved with increasing value and therefore that incentive-motivated enhancements in movement speed were not accompanied by tradeoffs in movement accuracy.

When comparing reward and punishment, we also found evidence for a positive interaction between value and valence ($\beta = 0.28, CI = \left[ { - 0.04, 0.61} \right], P_{d} = .05$), indicating that the value-driven enhancement of movement accuracy was more pronounced for Reward compared to Punishment. This was confirmed by fitting the model separately to data from each group, which showed that the value effect was greater for the Reward group ($\beta = 0.44, CI = \left[ {0.23, 0.64} \right], P_{d} < .001$), compared to the Punishment group ($\beta = 0.16, CI = \left[ { - 0.11, 0.43} \right], P_{d} = .12$). Lastly, a model-comparison analysis demonstrated that for Punishment data, a model with a binary value function predicted held-out data better than a model with a linear value function (binary – linear: $M{ } = - 10.4, SE = 6.57$) (Fig. 3A). However, for Reward data, a linear value function led to better predictive accuracy (linear – binary: $M{ } = - 12.6, SE = 6.93$). Consistent with our speed results, effects of increasing incentive value on movement accuracy were different for Reward and Punishment.

### Movement initiation improved with increasing incentive value

**Figure 4:**
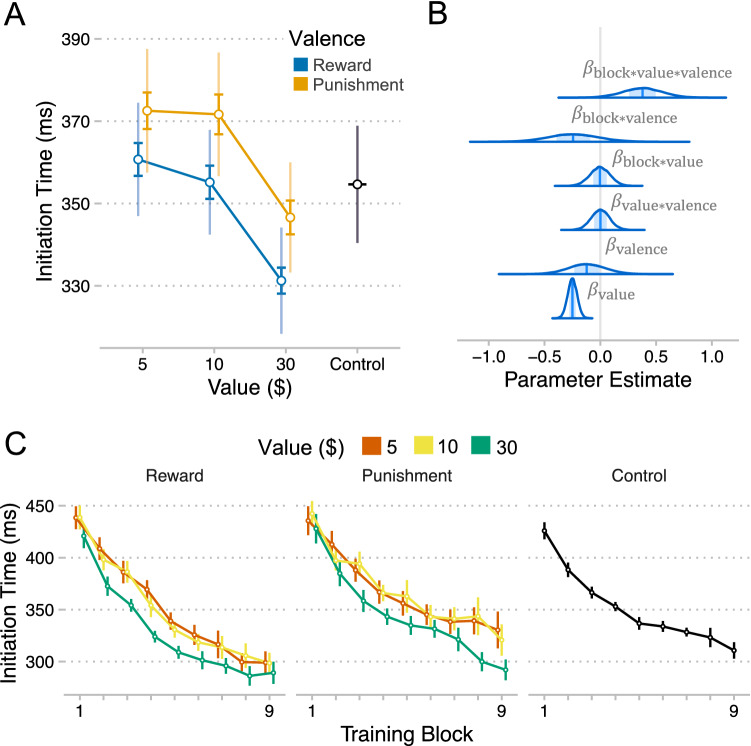
Effects of training incentives on movement initiation. (**A**) Initiation RT (mean + /- se) for each level of value by valence. The bold error bars represent within-subject standard error (for value effect) and the non-bold error bars are between-subject standard error (for valence effect). (**B**) Densities of MCMC samples used to approximate posterior probability distributions over key regression coefficients. (**C**) Initial RT for each level of block by value (sequence) by valence (group). Plots were created using ggplot2^46^.

Does training with incentives enhance the initiation of movement sequences? We addressed this question using a gaussian regression model of participants’ initial reaction times (RT) at each level of block by value by sequence (Fig. 4B). We measured RT as the duration between stimulus onset and the first response in the sequence (See Methods for modeling details). We found a negative linear effect of block on RT ($\beta = - 1.13, CI = \left[ { - 1.36, - 0.89} \right], P_{d} < .001$) and a positive quadratic effect of block ($\beta = 0.32, CI = \left[ {0.18, 0.45} \right], P_{d} < .001$), suggesting that participants initiated their responses more quickly over the course of training, albeit with diminishing returns (Fig. 2C). We found a negative effect of incentive value on RT, suggesting that response initiation was enhanced for high value sequences ($\beta = - 0.25, CI = \left[ { - 0.33, - 0.17} \right], P_{d} < .001$). RT in training was faster for large incentives compared to control irrespective of valence (Reward: $\beta = - 0.24, CI = \left[ { - 0.39, - 0.10} \right], P_{d} = .001$; Punishment: $\beta = - 0.08, CI = \left[ { - 0.23, 0.07} \right], P_{d} = .15$), but not so for small incentives (Reward: $\beta = 0.02, CI = \left[ { - 0.14, 0.17} \right], P_{d} = .41$; Punishment: $\beta = 0.16, CI = \left[ {0.00, 0.32} \right], P_{d} = .02$). We did not detect a main effect of valence ($\beta = - 0.12, CI = \left[ { - 0.45, 0.22} \right], P_{d} = .25$) or an interaction between block and valence ($\beta = - 0.23, CI = \left[ { - 0.67, 0.21} \right], P_{d} = .16$). However, model comparisons again showed that responses to punishment were more likely under binary value coding than linear value coding (binary – linear: $M{ } = - 6.66, SE = 5.19$), but we did not observe this difference for participants in the Reward group (binary – linear: $M{ } = - 0.13, SE = 5.65$) (Fig. 4A). This suggests that punishment values may be processed in a more categorical and less graded fashion compared to reward values.

### Value-driven impairment of performance retention

The results above show that incentives influenced performance during training. However, it remains to be seen whether these training incentives have a lasting influence on performance that persist when incentives are no longer available. We addressed this possibility by having participants return one day after training to perform the same sequences again without incentives. We applied the same regression models as above to response initiation, movement speed, and movement accuracy data at test.

**Figure 5:**
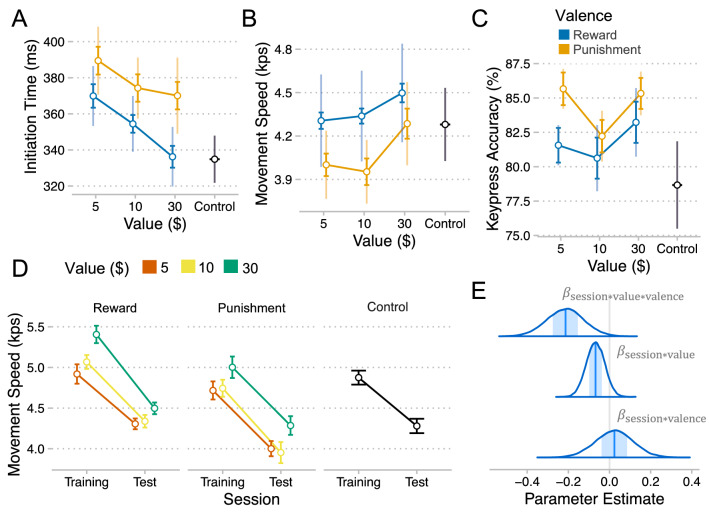
Effects of training incentives on performance retention. (**A**) Initial reaction time (mean + /- se) for each level of value by valence in the test. (**B**) Movement speed for each level of value by valence in test. (**C**) Keypress accuracy for each level of value by valence in test. (**D**) Change in speed from the end of training (blocks 7 – 9) to the retention test for each level of value by valence. E. Densities of MCMC samples used to approximate posterior probability distributions over key regression coefficients in our model of performance (MT) retention. Plots were created using ggplot2^46^.

The effect of value from training on response initiation also persisted to the test session, since there was a negative main effect of value on RT $\beta = - 0.23, CI = \left[ { - 0.39, - 0.08} \right], P_{d} = .003$) (Fig. 5A). Response initiation improved over the course of the test session, as evidenced by a negative linear effect of test block on RT ($\beta = - 0.11 CI = \left[ { - 0.22, 0.01} \right], P_{d} = .03$). We did not find evidence for other main effects or interactions in the model (all $P_{d}$ > 0.1).

The effect of value from training on movement speed persisted to the test session, since there was a positive main effect of value on speed ($\beta = 0.14, CI = \left[ {0.03, 0.25} \right], P_{d} = .02$) (Fig. 5B). Movement speed also improved over the course of the test session, as evidenced by a positive linear effect of test block on speed ($\beta = 0.27 CI = \left[ {0.19, 0.34} \right], P_{d} < .001$). We did not find evidence for other main effects or interactions in the model (all $P_{d}$ > 0.1).

While there was no main effect of prior value on movement accuracy during the test session ($$\beta = - 0.02, CI = \left[ { - 0.18, 0.14} \right], P_{d} = .40$$), we did find some evidence of a small negative main effect of valence ($$\beta = - 0.16, CI = \left[ { - 0.35, 0.04} \right], P_{d} = .06$$) (Fig. 5C). This appeared to be driven by especially low accuracy on sequences trained with $5 reward, as evidenced by a negative value by valence interaction ($$\beta = - 0.26, CI = \left[ { - 0.57, 0.04} \right], P_{d} = .05$$). Movement accuracy was relatively constant throughout the test session, as there was no effect of block ($$\beta = - 0.04 CI = \left[ { - 0.15, 0.07} \right], P_{d} = .22$$). We did not find evidence for other main effects or interactions in the model (all P > 0.1). Overall, it seems that the beneficial effects of value on accuracy did not persist into the test session and that training with small punishment may be more favorable for future movement accuracy than training with small reward.

The results above show that many of the beneficial effects of incentives on performance persisted to a test session the next day. But do incentives also protect against performance degradation over time? We address this question by comparing motor performance (movement speed, kps) at the end of training performance in the test session the following day (Fig. 5D, E) (See Methods for modeling details). We found evidence of decreased performance from training to the test session, as there was a negative effect of session on speed ($\beta = - 0.40, CI = \left[ { - 0.49, 0.31} \right], P_{d} < .001$). Critically, there was evidence of a negative interaction between session and value, suggesting that the decrease in speed from training to test was more pronounced for higher value sequences ($\beta = - 0.07, CI = \left[ { - 0.16, 0.02} \right], P_{d} = .06$) (Fig. 5D). Furthermore, there was a negative three-way interaction between session, value, and valence, which suggests that the value-driven impairment of performance retention was greater for Reward than Punishment ($\beta = - 0.21, CI = \left[ { - 0.40, - 0.04} \right], P_{d} = .01$). This was confirmed by fitting separate models to each group, which revealed a substantial negative effect of value on retention for the reward group (value*sess: $\beta = - 0.18, CI = \left[ { - 0.30, - 0.06} \right], P_{d} = .003$) but not in the punishment group ($\beta = 0.04, CI = \left[ { - 0.09, 0.18} \right], P_{d} = .26$). Other effects in this model were not significant ($P_{d}$ > 0.1). Overall, it appears that retention worsened with increasing incentive value, where again this incentive value effect was greater for reward than punishment, such that retention was worst for sequences paired with large rewards. However, it is unclear if this performance degradation is due to a loss of skill per se or rather a loss of motivation given that we did not measure performance on random sequences paired with incentives (see discussion).

## Discussion

Training to perform motor sequences with incentives led to enhancements of movement initiation, speed, and accuracy for sequences paired with large reward and these value effects persisted into the following day when participants were tested without incentives. Participants were unable to retain the level of performance they attained in training and retention was worst for motor sequences paired with high value reward. However, effects of increasing incentive value on motor on-line performance improvements were distinct for reward and punishment. Responses to punishment values showed a more binarized, categorical profile (large vs not) and responses to reward values showed a more linear profile. These results add nuance to prior work showing a dissociation between the effects of reward and punishment on motor skill learning.

We found that actions trained in association with large prospective incentives (+ /-$30) were performed and acquired more rapidly compared to sequences trained with small incentives (+ /-$5). A plausible explanation for this finding is that increasing incentive size increases the expected utility of success, thereby “paying the costs” associated with exerting more task-positive effort^10–12^. On this view, larger incentives make increased effort more worthwhile, and increased effort improves the performance and acquisition of skilled actions. When people are offered large reward, they are more willing to expend mental effort^13–15^ and physical effort^14,16,17^ and when people exert more effort, their performance and learning improve^18–21^. But sometimes conditions which enhance learning actually impair long-term retention^22^. Consistent with this we found that the performance levels for actions paired with larger reward were not retained as well as the performance levels for actions paired with smaller rewards.

We found that actions trained in association with rewards (i.e., monetary gains) were acquired better than actions trained with punishments (i.e., monetary losses). Some studies have examined the differential effects of reward and punishment on skill learning and the results have been mixed. Training with reward feedback has been associated with improved learning in a serial reaction time (SRT) task^2^, improve long-term memory retention in a force-tracking task^23^, and slower forgetting in a visuomotor adaptation task^24^. On the other hand, it has been shown that punishment leads to faster overall movement times during training in the SRT task^8^ and that training with punishment leads to faster on-line learning in a visuomotor adaptation task^24^. It appears that the effects of punishment and reward on skill learning depend on which task is being performed. As these tasks differ on the precise perceptual, cognitive, and motor demands placed on participants, it would be valuable to carefully determine how each of these processes is impacted by motivation.

Some previously observed effects of incentive valence on motor skill learning could have been due to loss aversion: perhaps punishment and reward had different effects because losses had greater subjective (dis)utility than gains^9^. Consistent with this view, we found that the effect of increasing incentive *value* resembled the effects of punishment (compared to reward) reported in prior work—namely, enhanced on-line performance and impaired retention. We found evidence that $30 reward led to the best on-line performance but the worst retention, compared to punishments or smaller rewards. However, we did not find that punishment enhanced training performance over reward. If anything, our results suggest that the effects of increasing incentive value were more pronounced for reward than punishment. Thus, it is unlikely that loss aversion can explain the differential effects of punishment and reward on motor learning.

The differential effects of punishment and reward on skill learning could arise from differences in the way punishment and reward are processed by the brain that are unrelated to loss aversion. For example, we found evidence that, in terms of their effects on behavior, rewards were encoded linearly while punishments were encoded in a binary manner. This encoding difference could be grounded in one of the many differential effects of punishment and reward on brain areas (e.g., orbital frontal cortex^1^, dorsal striatum^25^, ventral striatum^2^, ventral tegmental area^26^, hypothalamus^27^, medial prefrontal cortex^28^, insula^2^, and amygdala^27^) or pathways (e.g., dopaminergic^26^, GABAergic^26^ and serotonergic^29^). Future work should explore whether differences in the underlying neural mechanisms of reward and punishment processing can account for the differential effects of reward and punishment value on skill learning.

Consistent with prior work, we found that training with incentives, particularly large incentives, enhanced the on-line performance of action sequences compared to training with no incentives^2,8^. However, we found that training with incentives, particularly large rewards, was associated with a greater inability to maintain performance from training to test. This is consistent with the fact that conditions which foster rapid skill acquisition can impair long-term skill retention^22^. Studies have demonstrated several desirability difficulties^30^, such as varying practice conditions^31^, spacing study sessions^32^, and interleaving instruction^33^. Relatedly, it is possible that our control group benefitted from encoding specificity^34^ or transfer-appropriate processing^35,36^, whereas these beneficial context effects would be reduced for our incentive groups since there were no incentives in the test session. Furthermore, training without incentives may have encouraged intrinsic motivation, while training with incentives discouraged it, a phenomenon known as the undermining effect^37–39^. The undermining effect may have led participants in the incentive groups to be less motivated in the retention test session compared to participants in the non-incentive group. Future work would be required to disentangle these alternative accounts.

Two findings in the present study contradict the results of prior work and deserve extra attention. The first is that participants in the reward group improved more quickly during training than participants in the punishment group, whereas prior studies found that punishment enhanced online performance relative to reward^8,40^. However, a salient methodological difference between our study and prior studies is that we administered rewards and punishments prospectively with pre-trial cues, while prior work administered incentives retrospectively with feedback. Prospective and retrospective incentives may differentially engage error-based learning mechanisms^41^. For example, prospective incentive cues may bias learning to occur at the level of motor planning and action selection, while retrospective cues may bias learning to occur at the level of movement execution. While prior work has administered reward prospectively and found robust effects on the performance of learned skills, to our knowledge no prior study has examined the effects of prospectively administered punishment^3,42^. An interesting avenue for future research would be to directly compare the effects of prospective and retrospective incentives on skill learning and retention.

The second contradictory finding is that sequences paired with large reward showed larger performance decrements from training to retention test compared to sequences paired with no reward, whereas prior studies found that reward enhanced retention relative to control^2,4,40^. While our findings contradict the findings of the particular studies cited above, other studies have reported similar failures to replicate^8,43^. In fact, we expected the effects of large rewards to resemble the effects of punishment reported in prior work—namely, enhanced on-line performance and impaired off-line retention. The basis of this prediction is the hypothesis that the valence effects are driven by value differences vis a vis loss aversion. However, we note that our results directly comparing punishment to reward do not necessarily support this loss aversion hypothesis. It remains unclear what factors explain whether the prospect of reward enhances or impairs retention. Future studies could shed light on this puzzle by more precisely operationalizing skill retention so as to avoid potential confounds, as we discuss below.

In the present study, we operationalize learning as the improvement in performance with practice and we operationalize retention as the change in performance from the end of training to a short test the next day. We are limited by our experimental design in the specific conclusions we can draw about the memory representations involved in learning and retention. For example, it is unclear whether the changes in performance over time observed here are related to sequence-specific skill (e.g., 1–2-4–3-2–4-3–1), or a more general task skill (e.g., typing), or changes in motivation or arousal^44,45^. We think it is likely that all of these aspects are involved, but future studies are required to tease apart their relative importance. While it was not feasible here, given the already large number of conditions, future studies should administer untrained sequences during training and incentivized sequences during test to allow for all the comparisons necessary to adjudicate between these alternative explanations of the behavioral results.

In sum, we provide evidence that on-line motor performance was enhanced for sequences paired with large reward, while off-line performance retention was impaired. Effects of increasing incentive value were different for rewards and punishments, where sequences paired with $30 reward were performed the best during training but were retained the worst at test the next day. Whereas reward effects were linear, punishment effects tended to be binary. These findings add nuance to the growing literature on the effects of punishment and reward on skill learning by showing that incentive valence interacts with incentive value in complex and surprising ways.

## Methods

### Participants

93 undergraduate students at the University of Michigan participated in this experiment (Reward: N = 32, Punishment: N = 32, Control: N = 29).

### Ethics

All participants gave written informed consent to participate. Participants were compensated at a rate of ten U.S. dollars per hour plus cash bonuses. This study was approved by the University of Michigan IRB for Health Sciences and Behavioral Sciences and all methods were performed in accordance with the relevant guidelines and regulations. All software and data used in this manuscript will be made publicly available upon publication.

## Experiment

A diagram of the experimental protocol is shown in Fig. 1A. Participants completed a familiarization phase consisting of four blocks of 40 trials of the DSP task with random sequences. Next, they completed a training phase consisting of nine blocks of 36 trials of the DSP task with three cued sequences. For the Reward group, each sequence was paired with a $5, $10, or $30 reward; For the Punishment group, each sequence was paired with a -$5, -$10, or -$30 punishment; for the Control group, sequences were not paired with incentives. At the start of the experiment, participants in the Punishment group were endowed with $30 from which their potential losses would be deducted. During the training phase, success was based in part on a time limit that became stricter as the participant’s performance improved.

After the training phase, participants went home and did not practice for 24 h. After this day of rest, participants returned to the lab to complete a retention test consisting of three blocks of 40 trials. 30 of these trials were random sequences while 90 were the three sequences they trained on previously (30 trials each). At the end experiments for the incentive groups, one trial was selected at random from the training session. If the sequence was completed, without errors and faster than the time limit, the participant would gain or lose the amount associated with the sequence. Participants were given the option to receive payments at the end of each session, however everyone chose to receive all their payment at once at the end of the test session.

### Task

A diagram for an example of a successful DSP trial in the training is shown in Fig. 1B. Trials with cued sequences began with the presentation of a colored square associated with a unique sequence. For the incentive groups, the colored square was offset to the left and the incentive size was presented simultaneously in text to the right of the square (e.g., “$30”). The color cue and reward cue were redundant because there was a 1:1 mapping between sequence and incentive value (each sequence was paired with a unique value). For the control group, there was no incentive information presented and colored squares signifying sequence identity were presented centrally. Familiarization trials with random sequences began with a grey square. The cue period with sequence/reward information lasted for two seconds. Next, four gray squares are presented in a horizontal array and remained on the screen for one second. Then, one square changed color to white, indicating which of four keys the participant was to press with their left-hand (non-dominant) fingers (1: little finger, 2: ring, 3: middle, 4: index). Immediately after the correct key was pressed, the corresponding square changed color to black and a new square changed color to white, indicating the next key to press. This process repeated until the sequence was completed or a key-press error occurred. If a key-press error occurred, the most recently cued square changed color from white to red, and the trial was aborted after a 1 s delay. If the sequence was completed too slowly (i.e., completion took more time than allowed by the current time limit), then the stimuli disappeared and feedback text stating “Too slow” was presented centrally for 1 s. No additional feedback was given at the end of trials in which all eight key presses were completed within the time limit.

During the training session, each participant was constrained by a movement time limit that was dynamically updated to ensure that task difficulty was relatively stable within and between subjects. The time limit was defined as the median movement time on the previous twenty correct trials regardless of sequence. This time limit was used to determine whether the participant responded too slowly and hence whether they would gain or lose money if that trial were randomly selected at the end of the experiment (assuming there were no key-press errors).

### Incentive instructions

Participants in the Reward group were told that they would be playing to win a cash bonus of $5, $10, or $30, which would be displayed at the beginning of each trial. They were told that a colored square would indicate which sequence they must type and that each color would always be worth the same amount of money. They were encouraged to memorize these sequences as this would help them prepare, type faster, and win more bonus money. We clarified that they would not be accumulating money, but instead that at the end of the experiment, a trial would be chosen at random. If they correctly pressed all 8 buttons in time for that chosen trial, they would win the reward that was displayed for that trial; if they get it wrong, they will not win or lose any money.

Participants in the Punishment group were similarly told that they would be playing to win a cash bonus. We told them that they would be endowed with a $30 bonus at the outset of the experiment, but that they would have to perform well to avoid losing this money and that the penalty value would be displayed on each trial to tell them how much is at stake on that trial ($5, $10, or $30). They were encouraged to memorize these sequences as this will help them prepare, type faster, and avoid losing their $30 bonus. Finally, we clarified that If they correctly pressed all 8 buttons in time for the randomly chosen trial, they would keep their $30 bonus (plus their hourly wages of $20); if not, they will lose the amount of money that was displayed for that trial by subtracting it from their initial $30 bonus. Other instructions were identical to those of the Reward group.

### Data and statistics

Data visualization was performed using the R package ggplot2^46^. Error bars in the plots reflect within-subject standard errors, i.e., standard error of y – participant mean + grand mean^47^. Our plots use Wong’s color-scale, which was designed to be accessible to colorblind readers^48^.

Regression models were implemented using the R package brms: Bayesian Regression Models using Stan^49^ (v2.14.4; https://paul-buerkner.github.io/brms/). The backend of brms is the probabilistic programming language Stan, which uses Markov Chain Monte Carlo (MCMC) sampling to compute approximate posterior probability distributions for model parameters, such as regression coefficients^50^. We configured the MCMC sampler to run four chains in parallel with 4 k warmup and 6 k post-warmup iterations per chain. We assigned weakly informative default priors to all parameters (e.g., standard normal distributions for main effects and interactions)^51^. For each regression coefficient, we report the median estimate ($\beta$), the 95% credible interval^52^ ($CI$), and the proportion of the posterior with the wrong sign ($P_{d}$), equal to one minus the probability of direction (see this vignette for more on pd and its relation to the frequentist p-value: https://easystats.github.io/bayestestR/articles/probability_of_direction.html)^53^. To illustrate, we would summarize a Normal(1,1) posterior parameter estimate as $\beta = 1, CI = \left[ { - 0.96, 2.96} \right], P_{d} = .16$. Posterior summaries were obtained using the R-package bayestestR (v0.7.5; https://easystats.github.io/bayestestR/index.html).

Our analyses of training data focused on initial reaction time, movement speed, and keypress accuracy. We measured RT as the duration between stimulus onset and the first response in the sequence in milliseconds. We measured speed as the number of keys pressed (8) over movement time in keys per second (where movement time is the duration between the first and last key presses). Our analyses of RT and speed included only trials without keypress errors (i.e., complete sequences). For all three measures, speed, accuracy, and RT, we modeled means for each level of subject by value by valence by block, averaging over trials.

Our models of mean RT and speed used the gaussian response-function with density given by:

1 $$f\left( y \right) = \frac{1}{{\sqrt {2\pi } \sigma }}\exp \left( { - \frac{1}{2}\left( {\frac{y - \mu }{\sigma }} \right)^{2} } \right)$$

where $\sigma$ is the standard deviation of the residuals^54^. Our models of mean accuracy used a beta response-function with density for $y \in \left( {0,1} \right)$ given by:

2 $$f\left( y \right) = \frac{{y^{\mu \phi - 1} \left( {1 - y} \right)^{{\left( {1 - \mu } \right)\phi - 1}} }}{{B\left( {\mu \phi ,\left( {1 - \mu } \right)\phi } \right)}}$$

where $B$ is the beta function and $\phi$ is a positive precision parameter^54^. Since our accuracy data could also take on values *at* zero and one, we used a zero–one-inflated version of the beta family with density given by:

3 $$f_{\alpha ,\gamma } \left( y \right) = \begin{cases} \alpha \left( {1 - \gamma } \right) & \text{if y = 0,} \\ \alpha \gamma & \text{if y = 1,} \\ \left( {1 - \alpha } \right)f\left( y \right) & \text{otherwise.} \end{cases}$$

where $\alpha$ is the probability of zero or one and $\gamma$ is the probability of one but not zero^54^. All models included fixed effects of value, valence, linear block, quadratic block, linear block by value interaction, value by valence interaction, and linear block by value by valence interaction. Additionally, intercepts, and main effects of block and value were allowed to co-vary across participants around a multivariate population mean (i.e., the model allowed for ‘random effects’ of participant). Value (5, 10, 30) and valence (Punishment, Reward) were coded using linear contrasts (-0.5, 0, 0.5) and block was coded using orthogonal 2^nd^ degree polynomial contrasts. RT and speed were z-scored prior to modeling. Overall, our RT and speed models were specified as:

$$\begin{gathered} y_{i} { }\sim{\text{ normal}}\left( {\mu_{i} ,\sigma } \right) \hfill \\ \mu_{i} = \beta_{j} X_{i} \hfill \\ \beta_{j} \sim \beta + {\Sigma }Lb_{j} \hfill \\ \beta ,b_{j} \sim {\text{normal}}\left( {0,1} \right) \hfill \\ \sigma ,{\Sigma } \sim {\text{student}}\left( {3, 0, 2.5} \right) \hfill \\ L \sim {\text{lkj}}\_{\text{corr}}\_{\text{cholesky}}\left( 1 \right) \hfill \\ \end{gathered}$$

and our accuracy model was specified as:

$$\begin{gathered} y_{i} { }\sim{\text{ beta}}_{z} \left( {\mu_{i} ,\phi ,\alpha ,\gamma } \right) \hfill \\ {\text{logit}}\left( {\mu_{i} } \right) = \beta_{j} *X_{i} \hfill \\ \beta_{j} \sim \beta + {\Sigma }*L*b_{j} \hfill \\ \beta ,b_{j} \sim {\text{normal}}\left( {0,1} \right) \hfill \\ {\Sigma } \sim {\text{student}}\left( {3, 0, 2.5} \right) \hfill \\ L \sim {\text{lkj}}\_{\text{corr}}\_{\text{cholesky}}\left( 1 \right) \hfill \\ \alpha ,\gamma \sim {\text{beta}}\left( {1,1} \right) \hfill \\ \phi \sim {\text{gamma}}\left( {0.01,0.01} \right) \hfill \\ \end{gathered}$$

In the specifications above, $i$ denotes a particular trial and $j$ denotes a particular subject. $X_{i}$ are the predictors for a particular trial, $\beta_{j}$ are the regression coefficients for a particular subject, $\beta$ are the group mean regression coefficients, ${\Sigma }$ are the standard deviations of the coefficients across subjects, $L$ represents the correlation of coefficients across subjects, and $b_{j}$ are the random effects of subject on the regression coefficients. Note that this hierarchical parameterization was only applied to the within-subject main effects; for interactions and valence effects, $\beta_{j} = \beta$.

We also examined whether the value-response function was different for the two valence groups. We assessed this by comparing models that differed solely in the contrast used to encode value: linear (-0.5, 0, 0.5) or binary (-0.25, -0.25, 0.5). In this case, the models were estimated and compared separately for each group. We compared models using an approximate leave-one-out cross validation (LOO-CV) score intended to estimate the model’s expected log predictive density (ELPD) for out-of-sample data^55^. The theoretical ELPD for a new dataset is defined in Vehtari et al. (2017) as:

4 $$ELPD = \mathop \sum \limits_{i = 1}^{n} \smallint p_{t} \left( {\tilde{y}_{i} } \right)\log p\left( {\tilde{y}_{i} |y} \right)d\tilde{y}_{i}$$

where the $p(\tilde{y}_{i} )$’s are unknown quantities which represent the true generative process for $\tilde{y}_{i}$ and which can be approximated using cross-validation. In place of the true theoretical ELPD defined above, we use a leave-one-trial-out Bayesian estimate thereof, defined in Vehtari et al. (2017) as:

5 $$ELPD_{loo} = \mathop \sum \limits_{i = 1}^{n} \log p\left( {y_{i} |y_{ - i} } \right)$$

where

6 $$p\left( {y_{i} |y_{ - i} } \right) = \smallint p\left( {y_{i} {|}\theta } \right)p\left( {\theta {|}y_{ - i} } \right)d\theta$$

is the leave-one-out predictive density given the data sans the ith point. When the absolute mean difference in ELPD between two models exceeds the standard error of the differences (Eq. 23 in Vehtari et al. (2017), then the out-of-sample data are predicted better by the model with lower ELPD. We implement this model comparison analysis using the R package loo (v2.3.1; https://mc-stan.org/loo/index.html).

We fit a separate set of regression models to data from the test session, including RT, speed, and accuracy. These models included the same predictors as listed above. We also directly compared performance at the end of training (last 3 blocks) to performance in the test session (first 3 blocks) using a regression model of mean movement speeds at each level of block by session by value by valence. This regression model differed from those described above only in the inclusion of the session predictor (sesh) and its interactions with the other predictors: sesh*value, sesh*valence, block*sesh, block*sesh*value, block*sesh*valence, block* sesh*value*valence. While we were primarily interested in the effects involving session (e.g., sesh*value*valence), we included other predictors to ensure they are not driving the effects of interest. Intercepts and the main effects of block, session, and value were allowed to co-vary across participants around a multivariate population mean.

Lastly, we compared the performance of participants in our incentive groups to the performance of participants in our control group. We perform comparisons to control at the high and low levels of value ($5, $30) by valence (reward, punishment). Comparisons were performed using regression models which differed from our original models in that the value and valence predictors were replaced with a group predictor (incentive vs control) and there were no random effects.

## Data Availability

Data and code used for this project can be accessed at https://github.com/adkinsty.

## References

[1] O’Doherty J, Kringelbach ML, Rolls ET, Hornak J, Andrews C (2001). Abstract reward and punishment representations in the human orbitofrontal cortex. Nat. Neurosci..

[2] Wächter T, Lungu OV, Liu T, Willingham DT, Ashe J (2009). Differential effect of reward and punishment on procedural learning. J. Neurosci..

[3] Anderson SP, Adkins TJ, Gary BS, Lee TG (2020). Rewards interact with explicit knowledge to enhance skilled motor performance. J. Neurophysiol..

[4] Abe M (2011). Reward improves long-term retention of a motor memory through induction of offline memory gains. Curr. Biol..

[5] Adkins, T. J., Lewis, R. L., Lee, T. G. Large prospective losses lead to sub-optimal sensorimotor decisions in humans. *Biorxiv* (2020) doi: 10.1101/406439.

[6] Adkins, T. J. & Lee, T. G. Prospective rewards enhance motor skill performance and skill coding in LPFC. *Biorxiv* 2020.01.15.907006 (2020) doi:10.1101/2020.01.15.907006.

[7] Chen X, Holland P, Galea JM (2018). The effects of reward and punishment on motor skill learning. Curr. Opin. Behav. Sci..

[8] Steel A, Silson EH, Stagg CJ, Baker CI (2016). The impact of reward and punishment on skill learning depends on task demands. Sci Rep-uk.

[9] Tversky, A. & Kahneman, D. Prospect theory: An analysis of decision under risk. *Econometrica* **47**, (1979).

[10] Shenhav A, Botvinick MM, Cohen JD (2013). The expected value of control: an integrative theory of anterior cingulate cortex function. Neuron.

[11] Manohar SG (2015). Reward pays the cost of noise reduction in motor and cognitive control. Current Biol CB.

[12] Kool W, Botvinick M (2018). Mental labour. Nat Hum Behav.

[13] Hübner R, Schlösser J (2010). Monetary reward increases attentional effort in the flanker task. Psychon B Rev.

[14] Schmidt L, Lebreton M, Cléry-Melin M-L, Daunizeau J, Pessiglione M (2012). Neural mechanisms underlying motivation of mental versus physical effort. PLoS Biol..

[15] Westbrook J, Braver TS (2013). The economics of cognitive effort. Behav Brain Sci.

[16] Schmidt L (2008). Disconnecting force from money: effects of basal ganglia damage on incentive motivation. Brain.

[17] Chong TT-J (2015). Dopamine enhances willingness to exert effort for reward in Parkinson’s disease. Cortex.

[18] Craik FIM, Lockhart RS (1972). Levels of processing: a framework for memory research. J. Verb. Learn Verb. Be.

[19] Kahneman, D. *Attention and Effort*. vol. 1063 (Prentice-Hall, 1973).

[20] Craik FIM, Tulving E (1975). Depth of processing and the retention of words in episodic memory. J. Exp. Psychol. Gen.

[21] Lee TD, Swinnen SP, Serrien DJ (1994). Cognitive effort and motor learning. Quest.

[22] Schmidt RA, Bjork RA (1992). New conceptualizations of practice: common principles in three paradigms suggest new concepts for training. Psychol. Sci..

[23] Abe M (2011). Reward improves long-term retention of a motor memory through induction of offline memory gains. Curr. Biol..

[24] Galea, J. M., Mallia, E., Rothwell, J. & Diedrichsen, J. The dissociable effects of punishment and reward on motor learning. *Nat. Neurosci.* **18**, nn.3956 (2015). 10.1038/nn.3956 25706473

[25] Delgado MR, Locke HM, Stenger VA, Fiez JA (2003). Dorsal striatum responses to reward and punishment: effects of valence and magnitude manipulations. Cognit. Affect Behav. Neurosci..

[26] Cohen JY, Haesler S, Vong L, Lowell BB, Uchida N (2012). Neuron-type-specific signals for reward and punishment in the ventral tegmental area. Nature.

[27] Breiter HC, Aharon I, Kahneman D, Dale A, Shizgal P (2001). Functional imaging of neural responses to expectancy and experience of monetary gains and losses. Neuron.

[28] Gehring WJ, Willoughby AR (2002). The medial frontal cortex and the rapid processing of monetary gains and losses. Science.

[29] Cohen JY, Amoroso MW, Uchida N (2015). Serotonergic neurons signal reward and punishment on multiple timescales. Elife.

[30] Bjork, E. L. & Bjork, R. A. Making things hard on yourself, but in a good way: creating desirable difficulties to enhance learning using testing to improve learning and memory. in *Psychology and the Real World: Essays Illustrating Fundamental Contributions to Society* (eds. Gernsbacher, M. A., Pew, R. W., Hough, L. M. & Pomerantz, J. R.) vol. 2 59–68 (Worth Publishers, 2011).

[31] Smith SM, Glenberg A, Bjork RA (1978). Environmental context and human memory. Mem. Cognit..

[32] Bjork RA, Allen TW (1970). The spacing effect: consolidation or differential encoding?. J. Verb. Learn Verb. Be.

[33] Shea JB, Morgan RL (1979). Contextual interference effects on the acquisition, retention, and transfer of a motor skill. J. Exp. Psychol. Hum Learn Mem..

[34] Tulving E, Thomson DM (1973). Encoding specificity and retrieval processes in episodic memory. Psychol. Rev..

[35] Morris CD, Bransford JD, Franks JJ (1977). Levels of processing versus transfer appropriate processing. J. Verb. Learn Verb Be.

[36] Bransford, J. D., Franks, J. J., Morris, C. D. & Stein, B. S. Some general constraints on learning and memory research. in *Levels of Processing in Human Memory (PLE: Memory)* (eds. Cermak, L. S. & Graik, F. I. M.) (Taylor & Francis Group, 2014).

[37] Deci EL, Koestner R, Ryan RM (1999). The undermining effect is a reality after all—Extrinsic rewards, task interest, and self-determination: Reply to Eisenberger, Pierce, and Cameron (1999) and Lepper, Henderlong, and Gingras (1999). Psychol. Bull..

[38] Murayama K, Matsumoto M, Izuma K, Matsumoto K (2010). Neural basis of the undermining effect of monetary reward on intrinsic motivation. Proc. Natl. Acad. Sci..

[39] Cerasoli CP, Nicklin JM, Ford MT (2014). Intrinsic motivation and extrinsic incentives jointly predict performance: a 40-year meta-analysis. Psychol Bull.

[40] Galea JM, Mallia E, Rothwell J, Diedrichsen J (2015). The dissociable effects of punishment and reward on motor learning. Nat. Neurosci..

[41] Seidler RD, Kwak Y, Fling BW, Bernard JA (2013). Neurocognitive mechanisms of error-based motor learning. Adv. Exp. Med. Biol..

[42] Adkins TJ, Lee TG (2021). Reward modulates cortical representations of action. Neuroimage.

[43] Steel A, Baker CI, Stagg CJ (2020). Intention to learn modulates the impact of reward and punishment on sequence learning. Sci. Rep. UK.

[44] Robertson EM (2007). The serial reaction time task: implicit motor skill learning?. J. Neurosci..

[45] Branscheidt M (2019). Fatigue induces long-lasting detrimental changes in motor-skill learning. Elife.

[46] Wickham H (2016). ggplot2: Elegant Graphics for Data Analysis.

[47] Cousineau D (2005). Confidence intervals in within-subject designs: a simpler solution to Loftus and Masson’s method. Tutor. Quant. Methods Psychol..

[48] Wong B (2011). Points of view: color blindness. Nat Methods.

[49] Bürkner, P.-C. brms : An R Package for Bayesian Multilevel Models Using Stan. *J. Stat. Softw.* **80**, (2017).

[50] Carpenter, B. *et al.* Stan : a probabilistic programming language. *J. Stat. Softw.* **76**, (2017). 10.18637/jss.v076.i01 PMC9788645 36568334

[51] Gelman A, Jakulin A, Pittau MG, Su Y-S (2008). A weakly informative default prior distribution for logistic and other regression models. Ann. Appl. Stat..

[52] Kruschke, J. Doing bayesian data analysis: a tutorial with R, JAGS, and Stan. *Academic Press* (2014).

[53] Makowski D, Ben-Shachar MS, Chen SHAH, Lüdecke D (2019). Indices of effect existence and significance in the bayesian framework. Front. Psychol..

[54] Bürkner, P. Parameterization of response distributions in brms. *Vignette* https://cran.r-project.org/web/packages/brms/vignettes/brms_families.html (2020).

[55] Vehtari A, Gelman A, Gabry J (2017). Practical Bayesian model evaluation using leave-one-out cross-validation and WAIC. Stat. Comput..

